# Circumventing qPCR inhibition to amplify miRNAs in plasma

**DOI:** 10.1186/2050-7771-2-13

**Published:** 2014-07-22

**Authors:** Jordan L Plieskatt, Yanjun Feng, Gabriel Rinaldi, Jason P Mulvenna, Jeffrey M Bethony, Paul J Brindley

**Affiliations:** 1 Department of Microbiology, Immunology and Tropical Medicine, and Research Center for the Neglected Diseases of Poverty, School of Medicine and Health Sciences, George Washington University, Washington, D.C., USA; 2 Infectious Disease and Cancer, QIMR Berghofer Medical Research Institute, Brisbane, Queensland, Australia; 3 The University of Queensland, School of Biomedical Sciences, Brisbane, Queensland, Australia

**Keywords:** miRNA, Interference, Plasma, Reverse transcriptase, qPCR, Biomarker, Anti-coagulant, Heparin, Heparinase, Eliminase, *Bacteroides* heparinase I

## Abstract

**Background:**

Circulating microRNAs (c-miRNAs) have be identified in saliva, urine and blood, which has led to increasing interest in their development as biomarkers for diverse diseases including cancers. One of the key advantages of c-miRNAs over other biomarkers is the ability to be amplified and quantified by quantitative PCR (qPCR). However, at phlebotomy when whole blood is dispensed into heparinized tubes, residual levels of the anti-coagulant lithium heparin may remain in the plasma and hence with RNA isolated from the plasma. This can confound the detection of c-miRNAs by qPCR because it inhibits reverse transcriptase (RT). Here we present a procedure, modified from earlier techniques, to detect c-miRNAs in plasma that improves sensitivity and streamlines performance.

**Findings:**

Treatment of total RNA isolated from human blood plasma with *Bacteroides* heparinase I during reverse transcription at 37°C for one hour improved sensitivity and performance of the qPCR. This is in comparison to no treatment or treatment of the RNA prior to RT, which is the current suggested method and exposes plasma to *Flavobacterium heparinum* heparinase I for up to 2 hours before RT. This modest alteration improved qPCR performance and resulted in lowered threshold cycles (C_t_) for detection of the target sequence, candidate c-miRNA biomarkers, and controls. It also reduced the expense and number of processing steps, shortening the duration of the assay and minimizing exposure of RNA to elevated temperatures.

**Conclusion:**

Incorporating *Bacteroides* heparinase I treatment into conventional RT protocols targeting c-miRNA in plasma can be expected to expedite the discovery of biomarkers.

## Findings

Non-coding RNAs, including microRNAs (miRNAs), are increasingly the target of biomarker development [1]. These molecules play a central role in gene expression regulation, in particular at the posttranscriptional and homeostatic levels [2,3], and have been detected in specimen matrices used for cancer biomarker development, including solid tissues, urine, sera, and blood. [4-6]. Indeed, miRNAs have been developed as informative markers for breast [6], colorectal [7] and ovarian cancers [8,9]. As biomarkers, circulating miRNAs (c-miRNAs) may be preferable to miRNAs located in solid tumor tissues due to their accessibility, stability during storage, and the increased specificity and sensitivity of multiplexed assays i.e., approaches that allow the analysis of panels of c-miRNAs assembled together to identify miRNA signatures. Discovery of c-miRNAs biomarkers frequently begins with high throughput approaches such as microarrays or small RNA-Seq with subsequent verification by quantitative reverse transcription PCR (qPCR). The qPCR represents a confirmatory step that allows either absolute or relative quantitation of miRNAs expression, by integrating a standard curve or a reference control gene in the analysis, respectively [10].

In this short report we present a modified method for qPCR detection of c-miRNAs in plasma from human blood, collected using an anticoagulant, such as lithium heparin. Whereas most macromolecular constituents of plasma and serum are the same, and both are devoid of platelets, red and white blood cells, sera and plasma can be used interchangeably in serological analysis targeting antibodies or antigens [11]. However, plasma - unlike serum - retains the coagulation cascade clotting factors and fibrinogen, and problematically, exogenous anticoagulant(s) that block the blood from clotting. This presence of extraneous anticoagulants can interfere with the qPCR, including reverse transcription of the RNA to cDNA and amplification of the target cDNA [12-14]. Here we present an alternative to conventional approaches to overcome the inhibition from residual heparin in qPCR to detect c-miRNAs in plasma. These modifications to previously reported methods not only improved the qPCR but also reduced costs and streamlined the analysis by shortening processing time, and, thereby reduced the likelihood of RNA degradation.

Duplicate RT reactions were performed along with duplicate qPCRs for all the samples. The findings were compared with informative reports from others dealing with detection of c-miRNAs in sera. In ongoing studies, we plan to employ this modified protocol in discovery and validation of c-miRNAs as biomarkers for liver fluke induced cholangiocarcinoma (CCA) [15], with plasma collected in a longitudinal study and biobanked at -80°C. RNA was recovered from plasma (250 μl) using the QIAamp circulating nucleic acids kit (Qiagen) following the manufacturer’s standard protocol. In most cases, the RNA was reverse transcribed using the miScript II RT Kit (Qiagen) in HiSpec buffer and cDNA screened on custom miScript miRNA PCR Arrays (SA Biosciences). qPCR was performed with SYBR Green PCR Master Mix (miScript, Qiagen), a thermocycler (iCycler, Bio-Rad, Hercules, CA) fitted with real time detector (Bio-Rad iQ5), and the following thermal cycling: activation at 95°C, 15 min; 40 cycles of denaturation, 15 sec, 94°C, annealing, 30 sec 55°C, extension, 30 sec, 70°C); melting curve analysis, 81 cycles of 55°C, 20 sec dwell.

**Figure 1:**
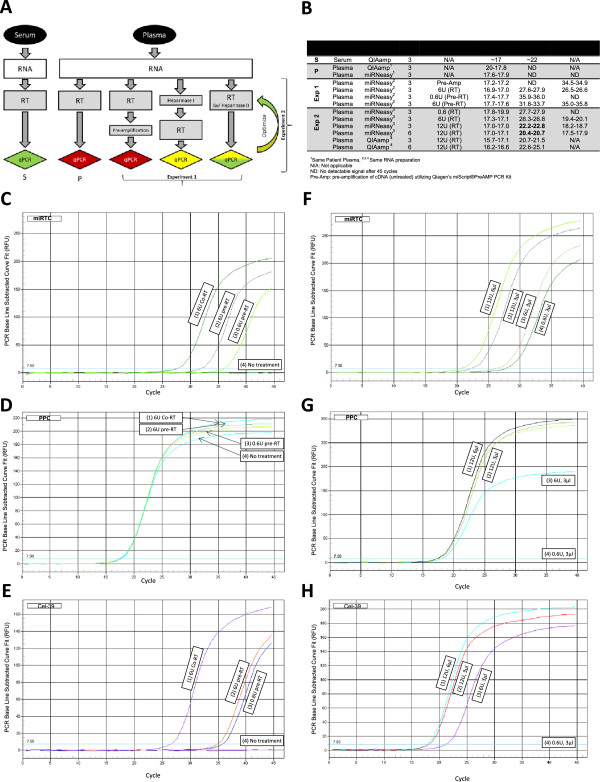
**Comparison of qRT-PCR controls obtained from RNA with and without treatment with *Bacteroides *heparinase I.** Panel **A**: Workflow of conditions tested for serum and plasma. Serum under standard conditions and plasma treated with *Bacteroides* heparinase I during the reverse transcription (RT) of RNA to cDNA yielded the most consistent signals. The colors indicate the level of performance – poor (red); fair (yellow); good (green). Panel **B**. Comparison of Ct values for qPCR controls obtained from RNA with and without treatment with *Bacteroides* heparinase I. Positive PCR Control (PPC) denoted a positive qPCR control and miRTC denotes reverse transcriptase controls. The plasma was spiked with *C. elegans* miR-39. Results (duplicate readings) for replicate samples for each treatment are shown. Optimization steps (Experiments 1 and 2) were carried out with plasma donated by the same persons. [Selected treatment conditions were confirmed using additional plasma (Figure 3, Additional file 1: Figure S1).] Panels **C-E**, Threshold cycles (C_t_ ) for qPCR controls with and without heparinase I. miRTC (panel **C**), PPC **(D)**, and miR-cel-39 **(E)** curves of Relative Fluorescent Units RFU (Y-axis) versus cycles (X-axis) for (left to right): 1) 6 U *Bacteroides* heparinase I during RT, 2) 6 U *Bacteroides* heparinase I treatment prior to RT, 3) 0.6 U *Bacteroides* heparinase I before RT, and 4) no treatment in duplicate (not visible along X-axis in Panel **C, E**). Panels **F-H**: miRTC curves of RFU (Y-axis) versus cycle time (X-axis) for (left to right): 1) 12 U, 6 μl; 2) 12 U, 3 μl; 3) 6 U, 3 μl; and 4) 0.6 U, 3 μl for Units of *Bacteroides* heparinase I and volume of RNA, respectively for miRTC (panel **F**), PPC **(G)** and miR-cel-39 **(H)**. Condition four yielded no curve for miR-cel-39 in panel **H** (presented on X-axis).

In contrast to studies using sera, miRNAs were not detectable from plasma (Figure 1A and B), including miRTC controls (miRTC: Reverse transcriptase qPCR; Qiagen) at 45 thermal cycles while PPC (Positive qPCR Controls, Qiagen) C_t_ values ranged from 18 and 20. To address this problem, we extracted RNA using the miRNeasy serum/blood plasma kit (Qiagen), which uses phenol and chloroform, and as a positive control, spiked plasma with *C. elegans* miR-39 mimic (Qiagen) [16]. The yield of RNA derived from plasma in both the QIAamp and miRNAeasy methods was 8-12 ng/μl, and was of similar quality to that we have recovered from sera [17], although RNA from plasma exhibited lower 260/230 ratios, (<0.7) with the miRNeasy kit. However, when RNA isolated using miRNeasy was subjected to qPCR, the C_t_ values did not improve, including for control *C. elegans* miR-39 which was employed to spike plasma (Figure 1A and B). This suggested interference, likely during reverse transcription; notably, the matrix of the original stored specimens - plasma versus serum - represented the only apparent difference from the previous analysis [17]. In reviewing the literature [12,13,18,19], a modified protocol was outlined for testing to overcome the residual heparin interference.

The first modification included in the approach for processing plasma (Experiment 1, Figure 1A and B) was replacement of *Flavobacterium heparinum* heparinase I with *Bacteroides* heparinase I (New England BioLabs) during RT (Experiment 1, Figure 1A and B). *Bacteroides* heparinase I cleaves 2-*O*-sulfated glucuronic acid residues in heparin in addition to the glycosidic bond between *N*-sulfated hexosamines and 2-*O*-sulfated iduronic acid residues cleaved by heparinase of *F. heparinum*[18,19]. Three reaction conditions were compared: (1) pre-treatment of RNA with *B.* heparinase I; (2) co-treatment of RNA during RT with *B.* heparinase I; and (3) pre-amplification. For pre-treatment of the RNA before RT [12,13], 0.6 U and 6.0 U *Bacteroides* heparinase I were incubated with 3 μl RNA for 2 h at 25°C then subsequently reverse transcribed. For co-treatment, the miScript II RT reaction (reaction volume, 20 μl) included 6 U *Bacteroides* heparinase I, 4 μl HiSpec 5× buffer, 2 μl 10× nucleic acids, 2 μl reverse transcriptase mix, and 3 μl RNA. For pre-amplification, the RT reaction was carried out according to the manufacturer’s protocol with the miScript Pre-AMP PCR Kit (Qiagen) for 12 cycles. In all cases, reverse transcription was carried out at 37°C for 1 h after which the RT was inactivated at 95°C, 5 min. The reaction products were diluted to 200 μl with water, stored on wet ice or at -80°C and analyzed by qPCR on miScript miRNA QA PCR Arrays (SA Biosciences). Minimal or no improvement was seen with the pre-amplification kit for the miRTC controls. By contrast, incubation with *Bacteroides* heparinase I before and during RT resulted in detectable miRTC values, with optimal amplification observed after inclusion of 6 U *Bacteroides* heparinase I during RT: miRTC, C_t_ ~28 (Figure 1B and C), PPC (Figure 1B and D), and miR-39, C_t_ ~26.5 (Figure 1B and E).

**Figure 2:**
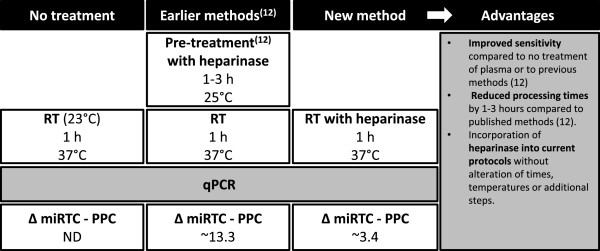
**Summary of advantages of the new approach concerning treatment of plasma with *Bacteroides *** **heparinase I before quantitative to detect c-miRNAs in plasma that improves sensitivity and streamlines performance.**

Because it eliminated one reaction step and thereby reduced exposure time of the RNA to elevated temperatures, co-treatment or inclusion of *Bacteroides* heparinase I during RT was examined, aiming for further improvement. Four conditions were tested: 0.6 U, 6 U and 12 U of *Bacteroides* heparinase I, with increasing quantities of RNA from 3 to 6 μl (Experiment 2; Figure 1A and B). Here C_t_ values were inversely related to the concentration of *Bacteroides* heparinase I (Figure 1B). Moreover, using 12 U *Bacteroides* heparinase I with 3 μl and 6 μl RNA resulted in miRTC values that were ≤ 5 cycles less than the positive qPCR controls (PPC) (Figure 1B, F, G and H). Last, to determine potentially negative influences of the RNA purification (QIAamp versus miRNeasy) on residual lithium heparin, RNA from QIAamp purifications (3 μl, 6 μl RNA) was also tested. In this case, QIAamp purifications were likely to have included more residual heparin since the plasma was not subjected to extraction in phenol-chloroform, and was in turn exposed to 12 U of *Bacteroides* heparinase I during RT. This condition yielded similar signals to matched samples treated as above (Figure 1B). To summarize, treatment with 12 U of *Bacteroides* heparinase I during RT at 37°C with 3 μl or 6 μl RNA delivered strong, positive and reproducible qPCR signals, markedly improving outcomes over no treatment or strategies similar to methods reported previously [12,13]. Additionally, co-treatment with *Bacteroides* heparinase I during RT offers the following advantages: 1) streamlines the protocol, without additional steps and reducing process times compared to earlier methods; 2) limits exposure of RNA to deleterious conditions including elevated temperatures for prolonged periods; and 3) improves sensitivity of qPCR compared to no-treatment or other approaches (Figure 2).

**Figure 3:**
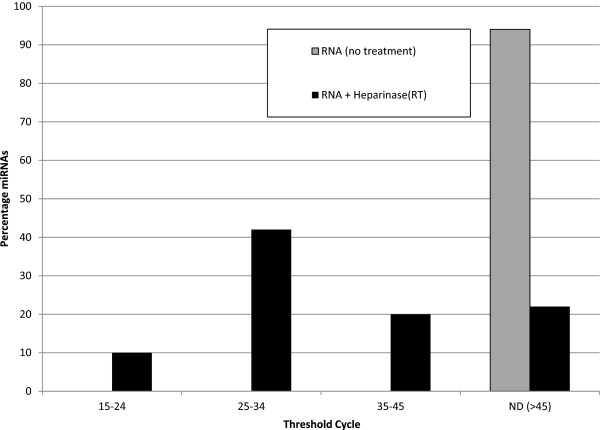
**Threshold cycles for qPCR targeting cholangiocarcinoma associated miRNAs in a larger panel of plasma samples.** Percentage of miRNAs based on their C_t_ values (not including the controls, PPC and RTC) from 19 samples of total RNAs isolated from plasma and not treated with *Bacteroides* heparinase I (no treatment) and with *Bacteroides* heparinase I during RT.

To confirm that this modified method for *Bacteroides* heparinase I improved sensitivity not only with assay and reaction controls, i.e., PPC, miRTC, miR-39, but could also be extrapolated to biomarker discovery, we examined the C_t_ results from a custom miScript miRNA PCR Array (SA Biosciences) across 19 discrete plasma samples. The arrays included 94 miRNA (mature, human) primers that provide potential signatures for cholangiocarcinoma [20]. We used 12 U *Bacteroides* heparinase I during RT and compared this with no-treatment of RNA. Without treatment, miRNAs remained undetected below 45 cycles on the custom miRNA array. By contrast, inclusion of *Bacteroides* heparinase I during RT significantly improved the sensitivity of detection: ~50% of the target miRNAs were detected at ≤ 35 cycles (Figure 3). Using this method, RNA from plasma was reproducibility reverse transcribed and amplified despite the presence of residual heparin in the plasma. Further, the miRTC and the ce-miR-39 spiked controls for 19 plasma samples from our investigation of biomarkers for CCA [20] are presented in Additional file 1: Figure S1. To conclude, the method presented here may be applicable to the analysis of miRNAs derived from blood, and should be especially advantageous for plasma from heparinized blood and/or where sera are unavailable.

### Ethics statement

Institutional Review Board of the George Washington University determined that the plasma samples used in this study did not meet the definition of human subjects research; i.e., a living individual about whom an investigator conducting research obtains: a) data through intervention or interaction with the individual or b) private identifiable information. This determination was made since the samples were limited to pre-existing, de-identified specimens labeled with a random code.

## Abbreviations

c-miRNAs: circulating miRNA (microRNA, micro-RNA); Ct: Threshold cycle; miRNA: microRNA, micro-RNA; miRTC: Reverse transcriptase qPCR controls (Qiagen); PPC: Positive qPCR Controls (Qiagen); qPCR: quantitative PCR (real-time PCR); RT: Reverse transcriptase and/or reverse transcription.

## Competing interests

The authors declare no competing interests.

## Authors’ contributions

JLP and YF carried out the assays; JLP optimized reaction conditions. JMB, GR, JPM, and PJB provided technical oversight and assisted in the preparation of the manuscript with JLP. All authors read and approved the final manuscript.

## Supplementary Material

Additional file 1: Figure S1 Box-and-whisker plot of threshold cycles (main axis) for miRTC and *C. elegans* miR-39 from 19 samples of threshold cycles exposed to *Bacteroides* heparinase I during the reverse transcription. Matched samples not treated with heparinase I failed to yield measurable C_t_ (>45), except for PPC. Click here for file
