# Supplementary figures and images for: Circumventing qPCR inhibition to amplify miRNAs in plasma
Source: Biomark Res. 2014 Jul 22;2:13. doi: 10.1186/2050-7771-2-13 (PMC4114091; doi:10.1186/2050-7771-2-13)

## Slide 1
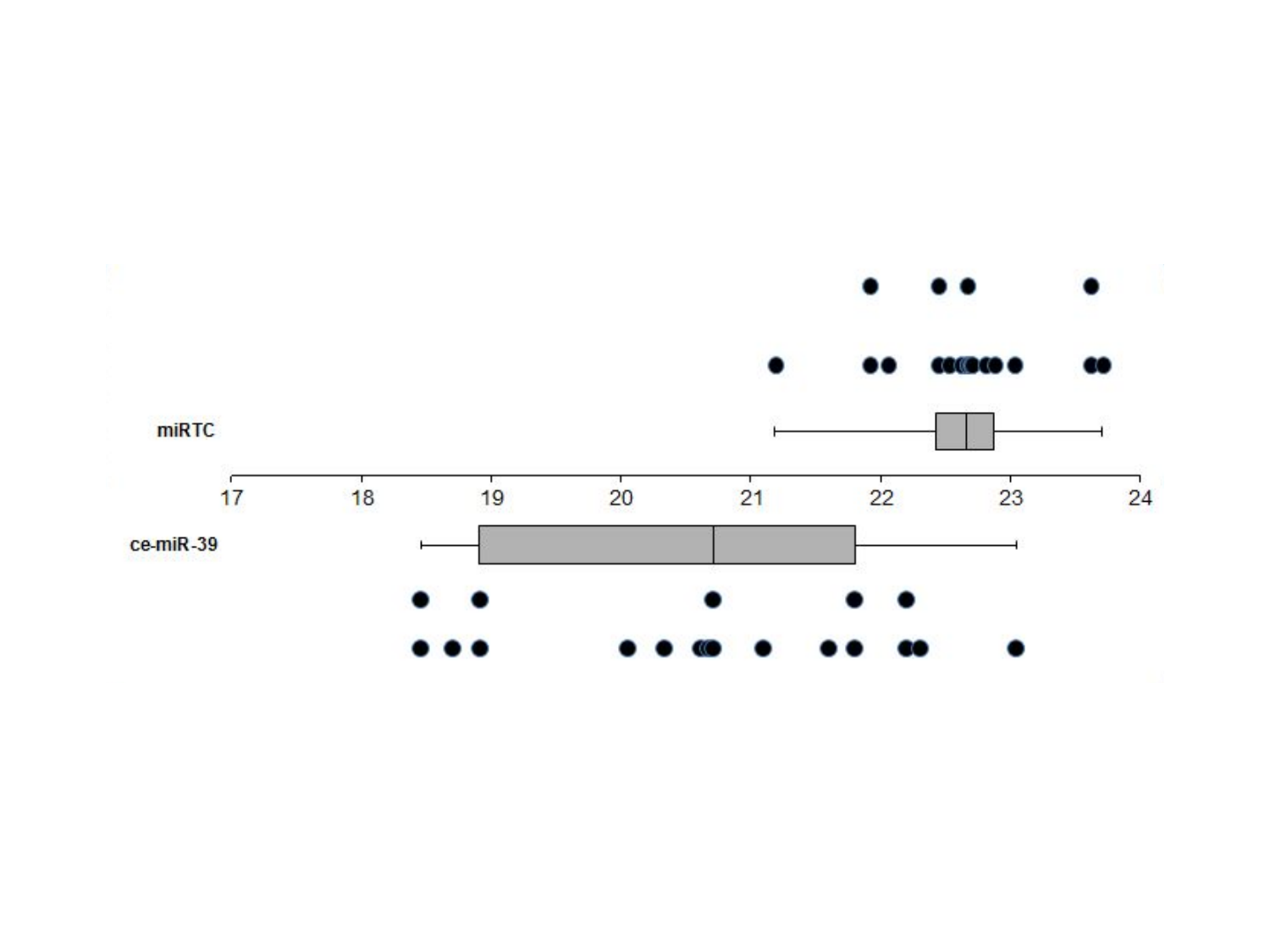

Supplement: Additional file 1: Figure S1 — Box-and-whisker plot of threshold cycles (main axis) for miRTC and C. elegans miR-39 from 19 samples of threshold cycles exposed to Bacteroides heparinase I during the reverse transcription. Matched samples not treated with heparinase I failed to yield measurable Ct (>45), except for PPC. [file 2050-7771-2-13-S1.pptx]
